# Supplementary material for: 1-Piperidine Propionic Acid Protects from Septic Shock Through Protease Receptor 2 Inhibition
Source: Int J Mol Sci. 2024 Oct 30;25(21):11662. doi: 10.3390/ijms252111662 (PMC11547144; doi:10.3390/ijms252111662)
Supplement: Supplementary file 1 [file ijms-25-11662-s001.zip › ijms-3163090-supplementary.pdf]

1-Piperidine Propionic Acid Protects from Septic Shock Through Protease Receptor 2 Inhibition

Roberto Luisetto [1,†], Marco Scarpa [1,†], Gianmarco Villano [1], Andrea Martini [2], Santina Quarta [3], Mariagrazia Ruvoletto [3], Pietro Guerra [3], Melania Scarpa [4], Monica Chinellato [3], Alessandra Biasiolo [3], Edoardo Campigotto [2], Daniela Basso [3], Matteo Fassan [3,5] and Patrizia Pontisso [3,*]

[1]Department of Surgical, Oncological and Gastroenterological Sciences, University of Padova, Via Giustiniani 2, 35128 Padova, Italy; roberto.luisetto@unipd.it (R.L.); marco.scarpa@unipd.it (M.S.); gianmarco.villano@unipd.it (G.V.)

[2]Department of Medicine, Azienda Ospedaliera-Università Padova, Via Giustiniani 2, 35128 Padova, Italy; andrea.martini@aopd.veneto.it (A.M.); edoardo.campigotto@aopd.veneto.it (E.C.)

[3]Department of Medicine, University of Padova, Via Giustiniani 2, 35128 Padova, Italy; santina.quarta@unipd.it (S.Q.); mariagrazia.ruvoletto@unipd.it (M.R.); pietro.guerra@studenti.unipd.it (P.G.); monica.chinellato@studenti.unipd.it (M.C.); alessandra.biasiolo@unipd.it (A.B.); daniela.basso@unipd.it (D.B.); matteo.fassan@unipd.it (M.F.)

[4]Immunology and Molecular Oncology Diagnostics, Veneto Institute of Oncology IOV-IRCCS, Via Gattamelata, 64, 35128 Padova, Italy; melania.scarpa@iov.veneto.it

[5]Veneto Institute of Oncology, IOV-IRCCS, Via Gattamelata, 64, 35128 Padova, Italy

* Correspondence: patrizia@unipd.it

† These authors contributed equally to this work.

SUPPLEMENTARY MATERIAL

SUPPLEMENTRY FIGURES

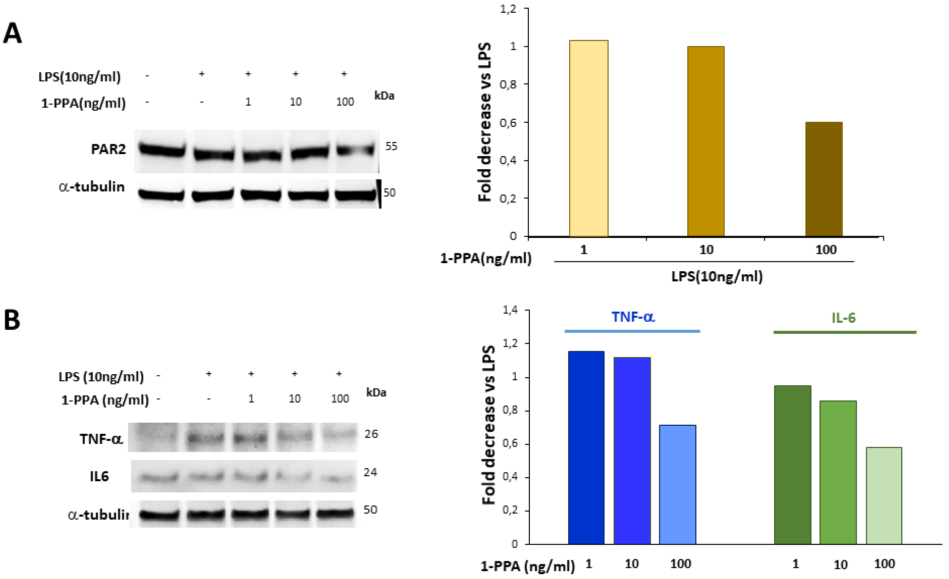

Suppl. Figure S1. Western Blot analysis of the effect of 1-piperidin propionic acid in LPS-treated THP-1 cell line. PAR2 protein expression (A) and TNF-α and IL-6 cytokine expression (B) in THP-1 cells treated for 5 hours with LPS (10 ng/ml) in absence or in presence of different concentrations of 1-piperidin propionic acid (1-PPA). In the right panels densitometric analysis of the corresponding Western blots is reported. Results are normalized to α-tubulin and expressed as fold decrease vs LPS.

A

	Body Condition Score[a]
**0**	Normal condition
**1**	Ruffling/coat condition
**2**	Ruffling/coat condition, decreased physical activity
**3**	Ruffling/coat condition, decreased physical activity, difficulty breathing
**4**	Ruffling/coat condition, decreased physical activity, difficulty breathing, abdomen moderately cold to the touch
**5**	Ruffling/coat condition, decreased physical activity, difficulty breathing, abdomen moderately cold to the touch, lethargy
**6**	Equivalent of death

[a]**Ullman-Culleré MH, Foltz CJ. Lab Anim Sci, 1999, 49: 319-323**

B

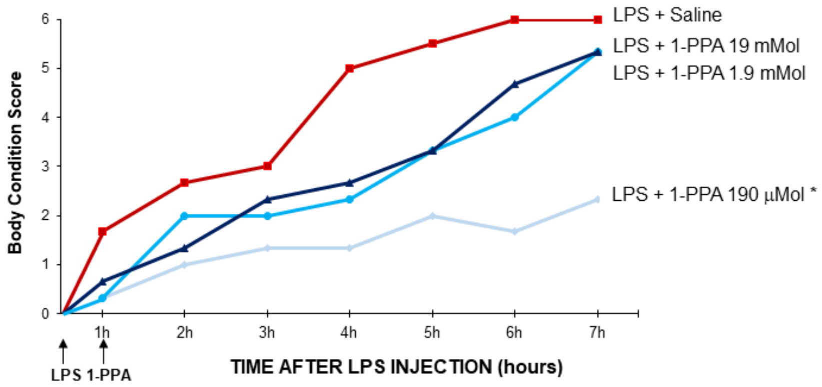

Suppl. Figure S2. Effect of different doses of 1-PPA on LPS-injected mice**. A**) Description of the Body Condition Score used to monitor mice symptoms. **B**) Monitoring of Body Condition Score in mice injected with LPS and treated with different concentrations of 1-PPA (190 mMol to 19 mMol) or saline solution (Saline) as control. Each point represents the mean score of the mice in different groups (N= 6/group). * p= 0.059 (Log-Rank Mantel Cox test).

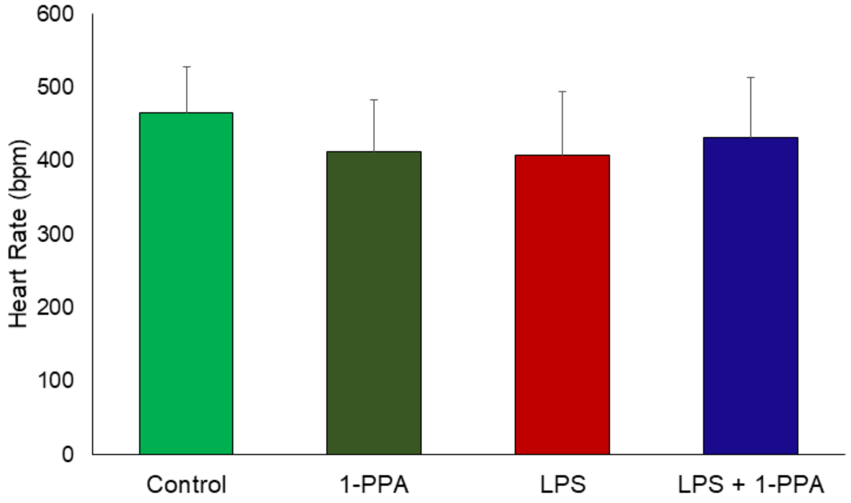

Suppl. Figure S3. Heart Rate in LPS-injected mice untreated or treated with 1-PPA. Heart Rate evaluation after 6 hours in mice injected with LPS and untreated or treated with 1-PPA at 190 mMol concentration (N=8/group). Additional control groups of mice (N=4/group) were not LPS-injected and not treated with 1-PPA (CONTROLS) or treated with 1-PPA without previous LPS injection (1-PPA). Data are expressed as median and interquartile range. No significant differences were observed between the different groups (Kruskal Wallis test).

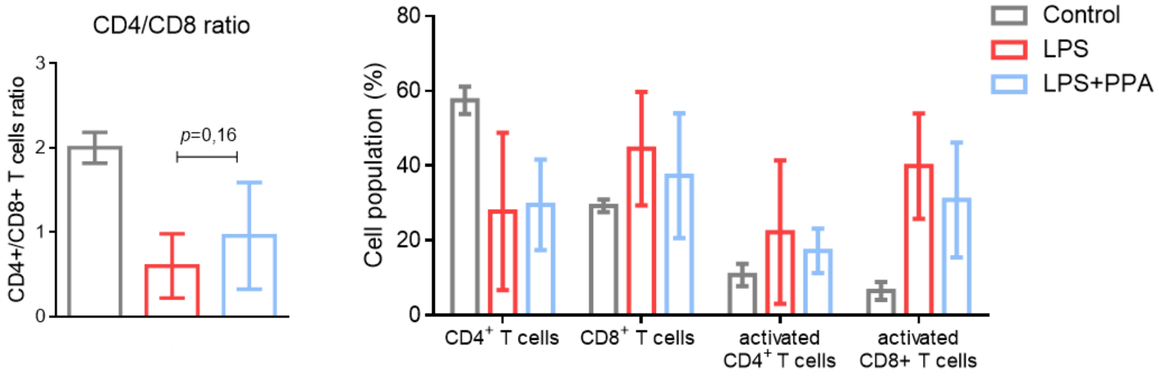

Suppl. Figure S4: Peritoneal fluid analysis**.** A) CD4/DC8 ratio in peritoneal fluid of control, LPS and LPS+ 1-PPA treatment groups (N= 7/group) after 6 hours of LPS injection. B) CD4+ and CD8+ total and activated T cells rate in the corresponding different groups of mice.

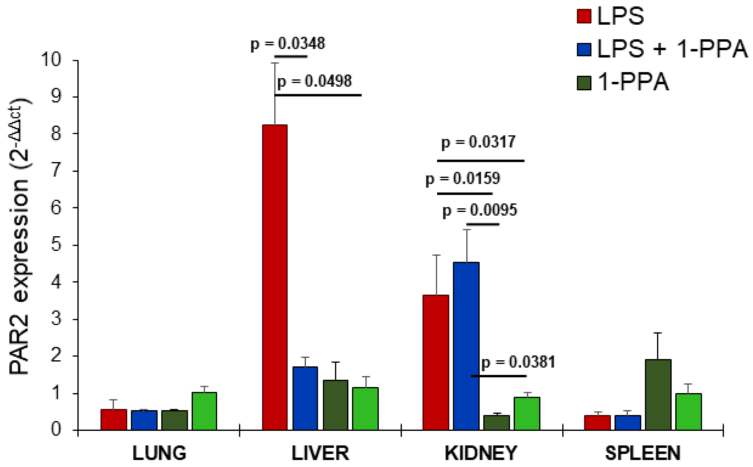

Suppl. Figure S5. PAR2 expression in different organs in LPS-injected mice untreated or treated with 1-PPA. Results are expressed as mean + SEM of gene expression, reported as 2-DDct relative to basal values. p values were reported only for significant differences, $p<0.05$ (Mann-Whitney test).

SUPPLEMENTAL TABLES

SUPPLEMENTAL TABLE S1. Threshold cycles stability of the housekeepers in untreated and LPS-treated conditions. The experiments were carried out in THP-1 cell lines for h-45s primers and in different organs for m-β-actin.

	Untreated	LPS treatment	p value
**Mean ± SEM of h-45s Cts**	12.50 ± 0.2301 N=7	12.60 ± 0.2439 N=7	0,7691
**Mean ± SEM of m-β-actin Cts**	24.28 ± 0.2460 N=14	24.45 ± 0.4603 N=18	0,768

Cts, threshold cycles; m, mouse; h, human

SUPPLEMENTAL TABLE S2. Description of antibodies used for FACS analysis of peritoneal fluid.

Antibody	Fluorophore	Clone	Company
CD45	APC	30-F11	eBioscience (Thermo Fisher Sci, Waltham, MA)
CD11b	PE	M1/70	eBioscience (Thermo Fisher Sci, Waltham, MA)
Ly6C	PE-Cy7	HK1.4	eBioscience (Thermo Fisher Sci, Waltham, MA)
Ly6G	FITC	1A8-Ly6g	eBioscience (Thermo Fisher Sci, Waltham, MA)
CD3	APC	17A2	eBioscience (Thermo Fisher Sci, Waltham, MA)

CD4	PE-Cy7	GK1.5	eBioscience (Thermo Fisher Sci, Waltham, MA)
CD8a	Alexa Fluor 488	53-6.7	eBioscience (Thermo Fisher Sci, Waltham, MA)
CD69	PE	H1.2F3	eBioscience (Thermo Fisher Sci, Waltham, MA)

SUPPLEMENTAL TABLE S3. List of the primers used in the study.

Target	Orientation	Sequence (5' to 3')
**m-β-actin**	Forward<br>Reverse	AGCCATGTACGTAGCCATCC<br>CTCTCAGCTGTGGTGGTGAA
**m-iNOS**	Forward<br>Reverse	GCATCCCAAGTACGAGTGGT<br>CCATGATGGTCACATTCTGC
**m-eNOS**	Forward<br>Reverse	ATCCAGTGCCCTGCTTCATC<br>GGCAGCCAAACACCAAAGTC
**m-IL-6**	Forward<br>Reverse	CCGGAGAGGAGACTTCACAG<br>TGGTCTTGGTCCTTAGCCAC
**m-Il-1β**	Forward<br>Reverse	GGAGAAGCTGTGGCAGCTA<br>GCTGATGTACCAGTTGGGGA
**m-TNF-α**	Forward<br>Reverse	GACCCTCACACTCAGATCAT<br>TTGAAGAGAACCTGGGAGTA
**m-CCL-2**	Forward<br>Reverse	GTCCCTGTCATGCTTCTGGG<br>GAAGACCTTAGGGCAGATGCAG
**m-BR1**	Forward<br>Reverse	TGTCCTTCTTCCTTTTGCCTTG<br>ACGACTTTGACGGAACGCAG
**m-BR2**	Forward<br>Reverse	GGTGCTGAGGAACAACGAGA<br>CAACACAGCACAAAGAGCCC
**m-KNG1**	Forward<br>Reverse	CCTTTGGAATGGTGATACCG<br>CGCAAATCTTGGTAGGTGGT
**h- IL1-β**	Forward<br>Reverse	TGAAAGCTCTCCACCTCCAG<br>CACGCAGGACAGGTACAGAT
**h- TNF-α**	Forward<br>Reverse	AACCTCCTCTCTGCCATCAA<br>GGAAGACCCCTCCCAGATAG
**h-IL-6**	Forward<br>Reverse	CTTCGGTCCAGTTGCCTTCT<br>TGTTTTCTGCCAGTGCCTCT

**h-TGF-β**	Forward<br>Reverse	AAGTGGACATCAACGGGTTC<br>GTCCTTGCGGAAGTCAATGT
**h-CCL2**	Forward<br>Reverse	CCCCAGTCACCTGCTGTTAT<br>AGATCTCCTTGGCCACAATG
**h-45s**	Forward<br>Reverse	CGGCTACCACATCCAAGGAA<br>GCTGGAATTACCGCGGCT

m, mouse; h, human
